# Identification of a fatty acid metabolism-related gene signature for prognostic prediction and immune microenvironment characterization in diffuse large B-cell lymphoma

**DOI:** 10.3389/fonc.2026.1798939

**Published:** 2026-04-10

**Authors:** Shuai Wang, Fuqiang Wang, Minjie Li, Zhimin Gu, Zhidong Gu

**Affiliations:** 1 Department of Laboratory Medicine, Ruijin Hospital, Shanghai Jiao Tong University School of Medicine, Shanghai, China; 2 College of Health Sciences and Technology, Shanghai Jiao Tong University School of Medicine, Shanghai, China; 3 State Key Laboratory of Common Mechanism Research for Major Diseases, Suzhou Institute of Systems Medicine, Chinese Academy of Medical Sciences & Peking Union Medical College, Suzhou, China; 4 Department of Laboratory Medicine, Ruijin-Hainan Hospital, Shanghai Jiao Tong University School of Medicine (Hainan Boao Research Hospital), Qionghai, China

**Keywords:** cancer immunity, diffuse large B-cell lymphoma, fatty acid metabolism, machine learning algorithms, prognosis prediction

## Abstract

**Background:**

Fatty acid metabolic reprogramming is critically implicated in tumorigenesis and progression. However, the role and prognostic significance of fatty acid metabolism-related genes (FMGs) in diffuse large B-cell lymphoma (DLBCL) remain largely unexplored.

**Methods:**

We analyzed transcriptomic data from the Gene Expression Omnibus (GEO) database to identify key prognostic FMGs. Through an integrative machine learning pipeline, we developed a prognostic signature termed the FAMscore. The association of the FAMscore with tumor immunity was assessed. Furthermore, we validated the dysregulation of multiple FMGs using quantitative real-time PCR and single-cell RNA sequencing data. Among these FMGs, we further investigated the role of CPT1A in DLBCL cell proliferation and apoptosis.

**Results:**

The FAMscore effectively distinguished between high- and low-risk DLBCL patients and served as an independent prognostic factor. A higher FAMscore was associated with poorer overall survival (OS). A nomogram integrating the cell-of-origin (COO) subtype, the International Prognostic Index (IPI) score, and the FAMscore was developed and demonstrated reliable predictive performance. Tumors in the high-FAMscore group exhibited higher tumor purity and an immune infiltration profile conducive to an immunosuppressive microenvironment. Functional assays revealed that knockdown of CPT1A significantly inhibited DLBCL cell proliferation and induced apoptosis.

**Conclusions:**

Our study highlights fatty acid metabolism as a key prognostic indicator and immune regulator in DLBCL. These findings advance the framework for personalized treatment strategies in this malignancy.

## 1 Introduction

Diffuse large B-cell lymphoma (DLBCL) is the most common pathological subtype of non-Hodgkin lymphoma, accounting for approximately 35–40% of cases (1, 2). It exhibits considerable biological and clinical heterogeneity (3, 4). DLBCL is highly aggressive and demonstrates rapid tumor growth and a propensity for early distant metastasis. The R-CHOP regimen is recognized as the standard first-line treatment, which can cure 60%–70% of patients and significantly improve overall survival (OS) (5). However, its therapeutic efficacy remains limited for patients with relapsed or refractory disease. Therefore, it is essential to establish reliable prognostic models for risk stratification and individualized management for DLBCL patients.

Cancer cells undergo metabolic reprogramming to acquire growth and survival advantages. Altered metabolism and its corresponding biological effects have become key features in understanding tumorigenesis, progression, and the underlying pathophysiological changes. Fatty acids, as essential components of lipids, play critical roles in energy supply, membrane synthesis, and signal transduction (6). Fatty acids esterify with glycerol to form triglycerides, which are stored as efficient energy reservoirs within lipid droplets of adipocytes. Under stress conditions such as starvation or exercise, triacylglycerols are hydrolyzed to release free fatty acids, which are subsequently mobilized as a fuel source for energy production (7). Fatty acids constitute the hydrophobic tails of phospholipids, forming indispensable structural elements of plasma membranes and organelle membranes. Furthermore, fatty acids and their derivatives, including prostaglandins and leukotrienes derived from arachidonic acid, act as essential signaling molecules that mediate various physiological and pathological processes such as inflammation, immune responses, and blood pressure regulation (8, 9).

Fatty acid metabolism (FAM), encompassing pathways of synthesis, elongation, activation, transport, and oxidation, has attracted increasing attention. Tumor cells often exhibit hyperactive fatty acid synthesis and/or β-oxidation, which facilitate proliferation, migration, stemness maintenance, and drug resistance (10, 11). Transformation of fatty acid metabolism also occurs in tumor-associated immune cells and stromal cells, which may contribute to an immunosuppressive and tumor-promoting microenvironment (12, 13). Studies have indicated that DLBCL highly relies on FAM (14–16), suggesting that targeting this metabolic pathway may represent a potential therapeutic strategy.

**Figure 1:**
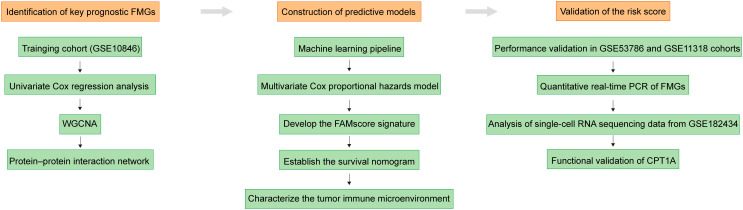
Workflow of this research.

In the present study, we systematically evaluated the prognostic value of fatty acid metabolism-related genes (FMGs) in DLBCL (**Figure 1**). By identifying key factors, a gene signature was constructed based on machine learning algorithms. We further assessed the predictive performance of this risk score and elucidated its association with the tumor immune microenvironment. Our findings may provide novel insights into the impact of fatty acid metabolism on clinical outcomes and the response to tumor immunotherapy in DLBCL.

## 2 Materials and methods

### 2.1 Data acquisition and preprocessing

RNA sequencing data and corresponding clinical information were obtained from the Gene Expression Omnibus (GEO) database. GSE10846 was utilized as the training cohort (17). Samples included in the research are those diagnosed as DLBCL, with complete clinicopathological information including age, gender, Ann Arbor stage, lactate dehydrogenase (LDH) levels, cell-of-origin (COO) classification, Eastern Cooperative Oncology Group (ECOG) performance status, number of extranodal involvement sites, and OS data. Additionally, the International Prognostic Index (IPI) was calculated based on the criteria defined by the International Non-Hodgkin’s Lymphoma Prognostic Factors Project (18). GSE53786 and GSE11318 cohorts were used for validation (19, 20), retaining samples with clearly documented age, gender, and OS information. Single-cell RNA sequencing data were sourced from the GSE182434 dataset, comprising four DLBCL samples and one pediatric tonsil sample (21).

FMGs were downloaded from the Human Molecular Signatures Database (MSigDB). We integrated relevant gene sets and collected a total of 622 genes (listed in **Supplementary Table S1**).

### 2.2 Identification of prognostic FMGs and consensus clustering

Univariate Cox regression analysis was conducted to identify FMGs associated with patients’ OS in the GSE10846 cohort. Genes with a p-value < 0.01 were defined as prognostic FMGs. Patients were clustered according to the expression of candidate FMGs using the ConsensusClusterPlus R package (22). The optimal number of clusters was determined through a combined examination of the consensus matrix and the cumulative distribution function (CDF) curve.

### 2.3 Weighted gene co-expression network analysis

To identify genes exhibiting highly synergistic expression patterns, we employed the WGCNA R package to analyze candidate prognostic FMGs (23). An appropriate soft threshold β was selected to construct an adjacency matrix, which was subsequently transformed into a topological overlap matrix (TOM). Module identification was performed using the dynamic tree cutting method. The correlations between module eigengenes (MEs) and clinical characteristics and consensus clusters were systematically evaluated.

### 2.4 Protein-protein interaction network

The PPI network for FMGs was constructed using the Search Tool for the Retrieval of Interacting Genes (STRING) database, with a minimum required interaction score threshold of 0.400 (default medium confidence). Cytoscape software (version 3.9.0) and the cytoHubba plugin were employed to analyze the PPI network, in which genes with a Maximal Clique Centrality (MCC) score greater than or equal to the median value were identified as key prognostic genes for subsequent analyses.

### 2.5 Construction of the gene signature through an integrative machine learning pipeline

To develop a prognostic model with high accuracy and stability, we integrated 10 machine learning algorithms, resulting in 101 algorithm combinations based on a previous study (24). The algorithms included Elastic Net (Enet), Lasso, Ridge, Stepwise Cox, CoxBoost, Random Survival Forest (RSF), Partial Least Squares Regression for Cox Models (plsRcox), Supervised Principal Component Analysis (SuperPC), Generalized Boosted Regression Modeling (GBM), and Survival Support Vector Machine (survival-SVM). The algorithm parameter settings are provided in **Supplementary Table S2**, and the model combinations are provided in **Supplementary Table S3**. These combinations were applied to the key prognostic FMGs to fit prediction models within a 10-fold cross-validation framework using the GSE10846 cohort. The models were subsequently validated in the GSE53786 and GSE11318 cohorts, and their performance was assessed using Harrell’ s concordance index (C-index). The model yielding the highest average C-index was identified as optimal.

### 2.6 Nomogram establishment

Univariable and multivariable Cox regression analyses were performed using the survival R package to determine the prognostic values of the FAMscore and various clinical features. The results were visualized by the forestplot R package. Based on the screened independent prognostic indicators, a nomogram was generated with the rms package to predict clinical outcomes in DLBCL patients. Moreover, the predictive performance of the nomogram was evaluated using the survminer, timeROC, rms, and ggDCA (25) R packages.

### 2.7 Estimation of the tumor immune microenvironment

To characterize the tumor microenvironment, the estimate R package was used to calculate the immune score, stromal score, and tumor purity of the DLBCL samples (26). Immune cell fractions were estimated using the CIBERSORT algorithm (version 1.0) (27), with source code obtained from https://cibersort.stanford.edu/. The analysis was performed using the LM22 signature matrix and 100 permutations. Quantile normalization (QN=TRUE) was applied to normalize expression distributions across samples. Furthermore, Spearman’s rank correlation analysis was conducted to assess the associations of the risk score and FMGs included in the gene signature with immune checkpoint expression.

### 2.8 Pathway enrichment analysis

Differential expression analysis was performed using the limma R package between the high- and low-risk score groups (28). Genes were sorted in descending order based on log2FC, and gene set enrichment analysis (GSEA) was conducted using the clusterProfiler R package to evaluate enrichment in the Hallmark gene set collection (h.all.v7.0.entrez.gmt) from the MSigDB database (29). Enrichment was quantified using the normalized enrichment score (NES), and statistical significance was evaluated with p-values and false discovery rate (FDR)-adjusted p-values.

### 2.9 Analysis of single-cell sequencing data

Quality control and preprocessing of single-cell sequencing data were performed using the Seurat package (version 4.4.0), which included data normalization, selection of variable features, scaling, dimensionality reduction via principal component analysis (PCA), and further nonlinear dimensionality reduction with UMAP (30). Cell type annotation was applied based on the original classifications provided by Steen et al. (21). Differential expression of FMGs between normal and tumor samples was visualized using the DotPlot function. The FAMscore for each cell was computed using the UCell R package (31).

### 2.10 Quantitative real-time PCR analysis

GM12878, Ri-1, and TMD-8 cells were cultured in RPMI 1640 medium supplemented with 10% fetal bovine serum (FBS) and 1% penicillin-streptomycin. Total RNA was extracted with TRIzol (#15596018CN; Invitrogen) and reverse transcribed using cDNA Synthesis SuperMix (#11141; Yeason). Quantitative PCR was carried out utilizing SYBR Green Master Mix (#11184; Yeason) on a QuantStudio^TM1^ Real-Time PCR System (ThermoFisher) following standard procedures. ACTB was employed as a reference gene, and the relative gene expression was calculated using the ΔΔCt method. Primer sequences are provided in **Supplementary Table S4**.

### 2.11 CPT1A knockdown

To generate CPT1A-knockdown cells, short hairpin RNAs (shRNAs) targeting CPT1A or a non-targeting control (shNC) were cloned into a pLKO.1 vector carrying a GFP reporter. Lentiviruses were then produced and used to transduce DLBCL cells. Successfully transduced cells, which co-expressed GFP, were subsequently enriched by fluorescence-activated cell sorting (FACS) to establish stable CPT1A-knockdown and control cell lines. The shRNA sequences used were as follows: shCPT1A#1, 5’-GCCATGAAGCTCTTAGACAAA-3’; shCPT1A#2, 5’-GGATGGGTATGGTCAAGATCT-3’; and the negative control shNC, 5’-CAACAAGATGAAGAGCACCAA-3’.

### 2.12 Cell proliferation assay

Cell proliferation was assessed using the Cell Counting Kit-8 (CCK-8) assay. Briefly, cells were seeded in 96-well plates at a density of 2 × 10³ cells per well in 100 µL of culture medium. Five replicate plates were prepared to allow for measurements at 0, 24, 48, 72, and 96 hours post-seeding. At each indicated time point, 10 µL of CCK-8 reagent (#40203ES; Yeason) was added to the corresponding wells. After incubation in the dark at 37 °C for 2.5 hours, the absorbance was measured at 450 nm (OD_450_) using an Agilent BioTek Synergy H1 microplate reader. Relative cell proliferation was determined by normalizing the absorbance of each time point to that of the 0-hour time point, after subtracting the background absorbance from medium-only controls.

### 2.13 Apoptosis analysis

For apoptosis analysis, cells were harvested and washed with ice-cold phosphate-buffered saline (PBS). The cells were then stained with Annexin V-PE (#40310ES; Yeason) and DAPI (#D10112; Psaitong) for 15 minutes at room temperature in the dark. Stained cells were immediately analyzed using a BD FACSymphony A1 flow cytometer to determine the percentage of apoptotic cells.

### 2.14 Statistical analysis

All sequencing data processing and statistical analyses were performed using R software (version 4.5.1). Experimental data analysis was conducted with GraphPad Prism software (version 8.0.1). Comparisons between two groups were performed using the Wilcoxon rank-sum test, whereas one-way or two-way analysis of variance (ANOVA) followed by Dunnett’ s multiple comparison test was applied for comparisons among multiple groups. Correlation analysis was carried out using Spearman’s method. Survival analysis was performed using the Kaplan-Meier method, and the log-rank Mantel-Cox test was used to compare survival curves between groups. P values less than 0.05 were considered statistically significant. *P < 0.05, **P < 0.01, ***P < 0.001, ****P < 0.0001.

## 3 Results

### 3.1 Identification of key prognostic FMGs in DLBCL

**Figure 2:**
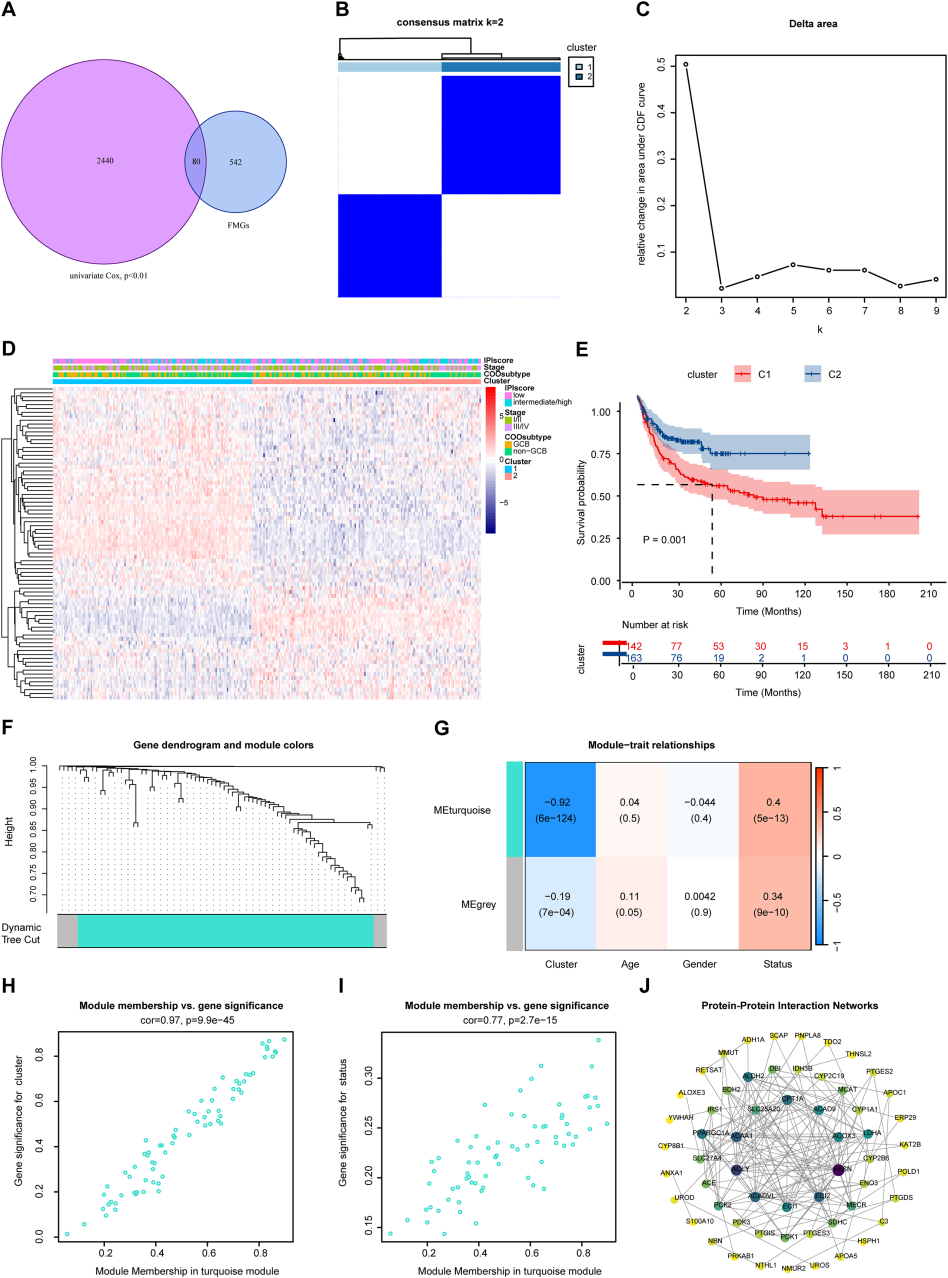
Identification of prognostic fatty acid metabolism-related genes in DLBCL. **(A)** Venn diagram illustrating the overlap between results from univariate Cox regression analysis in the GSE10846 cohort and the fatty acid metabolism-related gene set. **(B)** Consensus matrix heatmap at k = 2. For the optimal k value, the matrix exhibits clear and distinct block-like structures, indicating high clustering stability and strong within-cluster consistency. **(C)** Relative change in the area under the CDF curve for different k values. **(D)** Heatmap displaying the expression profiles of 80 fatty acid metabolism-related genes (FMGs) in patients from the two clusters. **(E)** Survival analysis comparing patients between the two clusters. **(F)** Gene dendrogram representing gene clustering in WGCNA modules. **(G)** Heatmap showing correlations between module eigengenes and clinical features. **(H)** Correlation between module membership in the turquoise module and gene significance for cluster assignment. **(I)** Correlation between module membership in the turquoise module and gene significance for OS status. **(J)** Protein-protein interaction network of genes in the turquoise module. Node color and line thickness represent the degree of connectivity. Genes located in the two central circles are identified as key prognostic FMGs.

To systematically identify FMGs associated with overall survival in DLBCL patients, we first conducted univariate Cox regression analysis in the GSE10846 cohort and identified 2520 genes with prognostic values (P < 0.01). By intersecting these with FMGs, we obtained 80 prognostic FMGs (**Figure 2A**). Consensus clustering was then applied to all patient samples based on the expression profiles of these 80 genes. The consensus matrix heatmap, CDF curve, and cluster consensus results indicated that the optimal clustering was achieved when samples were divided into two groups (**Figures 2B, C**, **Supplementary Figures 1A, B**). The heatmap revealed significant differences in the expression of prognostic FMGs and clinicopathological parameters between the two clusters (**Figure 2D**). Kaplan-Meier survival analysis demonstrated that patients in cluster 1 had a significantly poorer prognosis than those in cluster 2 (**Figure 2E**). Subsequently, we performed WGCNA on the candidate prognostic FMGs, setting the soft threshold β to 4. This identified a core co-expression turquoise module, comprising 72 genes, and a minor gray module containing 8 genes (**Figure 2F**, **Supplementary Figures 1C–E**). The turquoise module exhibited stronger correlations with both survival status and consensus clustering (**Figures 2G–I**). We then constructed a PPI network for the FMGs within this module and visualized it using Cytoscape. To prioritize key genes, we applied the Maximal Clique Centrality (MCC) algorithm, and those with scores above the median were selected as key prognostic FMGs for subsequent analysis (**Figure 2J**).

### 3.2 Construction of an FMG signature using machine learning algorithms

**Figure 3:**
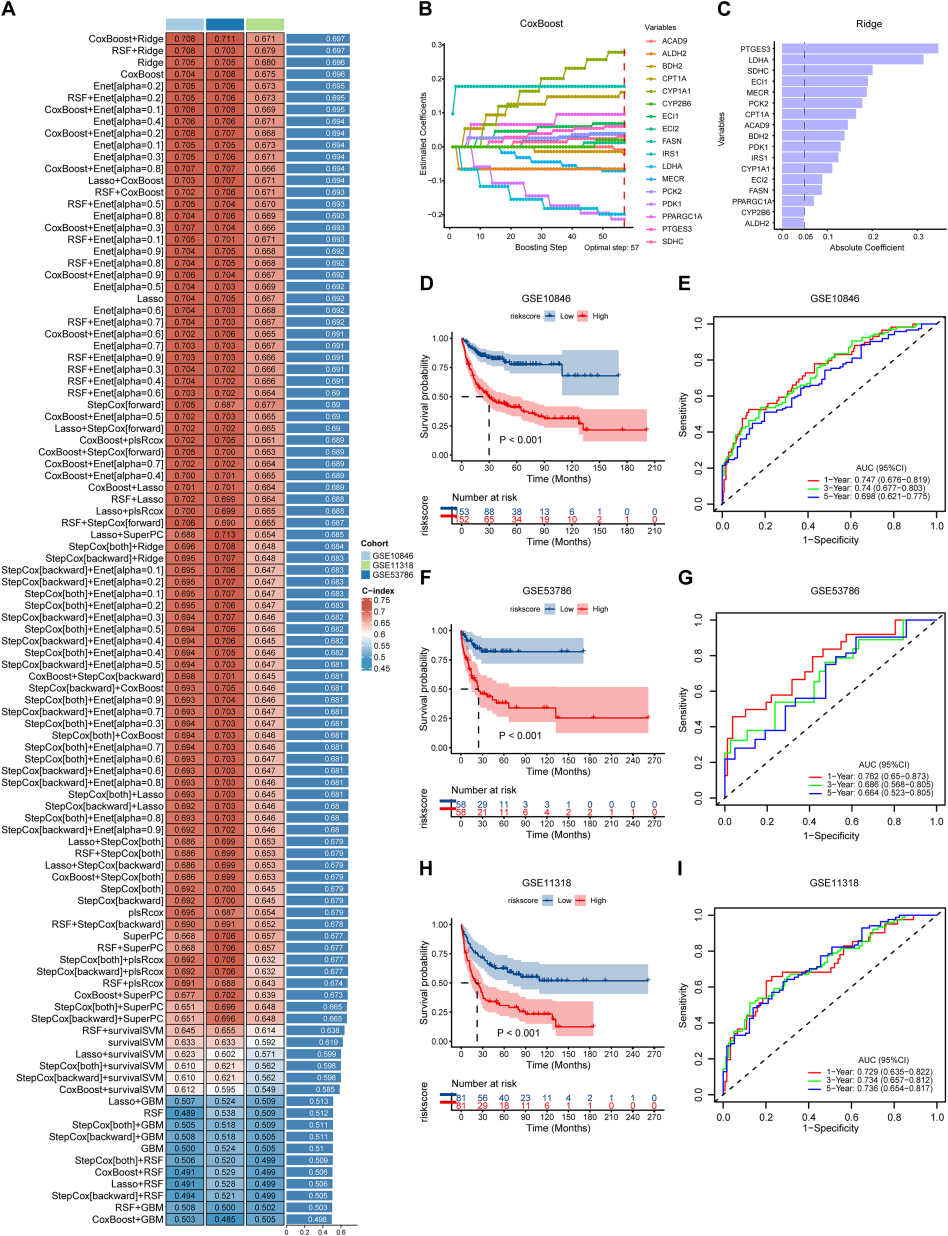
Construction of an FMG signature using machine learning algorithms. **(A)** Variable selection and model construction based on 31 key prognostic FMGs in the GSE10846 cohort, utilizing 101 algorithm combinations. **(B)** Estimated coefficients of the selected variables derived from the CoxBoost algorithm. **(C)** Absolute coefficients of variables in the Ridge algorithm, indicating the contribution of each FMG to the model’s predictive power. **(D, F, H)** Kaplan-Meier survival analysis of patients in the GSE10846 **(D)**, GSE53786 **(F)**, and GSE11318 **(H)** cohorts. **(E, G, I)** Time-dependent ROC analysis predicting 1-year, 3-year, and 5-year OS in the GSE10846 **(E)**, GSE53786 **(G)**, and GSE11318 **(I)** cohorts.

In the GSE10846 dataset, we fitted 101 prediction models based on the expression profiles of 31 FMGs utilizing a machine learning pipeline, and computed the concordance index (C-index) for each model in the training cohort and two validation cohorts (GSE53786 and GSE11318) (**Figure 3A****;** **Supplementary Table S5**). The optimal model was an ensemble of CoxBoost and Ridge, which achieved the highest mean C-index (0.697). The CoxBoost algorithm identified 17 variables with non-zero coefficients through 57 iterative steps (**Figure 3B**). These 17 FMGs were subsequently analyzed using the Ridge algorithm, which ranked them by relative importance and selected those with absolute coefficient values greater than 0.05 (**Figure 3C**). Ultimately, we identified 15 FMGs and used them to construct a multivariate Cox model, which generated a risk score for each patient, defined as the FAMscore. The formula is as follows: Risk score = (0.558 × PTGES3) + (-0.516 × LDHA) + (0.386 × SDHC) + (0.193 × ECI1) + (0.362 × MECR) + (0.231 × PCK2) + (0.266 × CPT1A) + (-0.548 × ACAD9) + (-0.162 × BDH2) + (-0.214 × PDK1) + (-0.173 × IRS1) + (0.161 × CYP1A1) + (0.066 × ECI2) + (0.176 × FASN) + (0.095 × PPARGC1A).

To validate the predictive performance of the risk score, patients were stratified into high- and low-FAMscore groups according to the median risk score. Kaplan-Meier survival analysis demonstrated that the high-FAMscore group had a significantly shorter overall survival compared to the low-FAMscore group (**Figure 3D**). Consistently, DLBCL patients in the high-FAMscore group exhibited lower survival rates in both the GSE53786 and GSE11318 cohorts (**Figures 3F, H**). Furthermore, receiver operating characteristic (ROC) curve analysis indicated that the risk score achieved high area under the curve (AUC) values for 1−, 3−, and 5−year survival across all three cohorts, demonstrating robust discriminative ability (**Figures 3E, G, I**).

### 3.3 The FAMscore demonstrates clinical relevance

**Figure 4:**
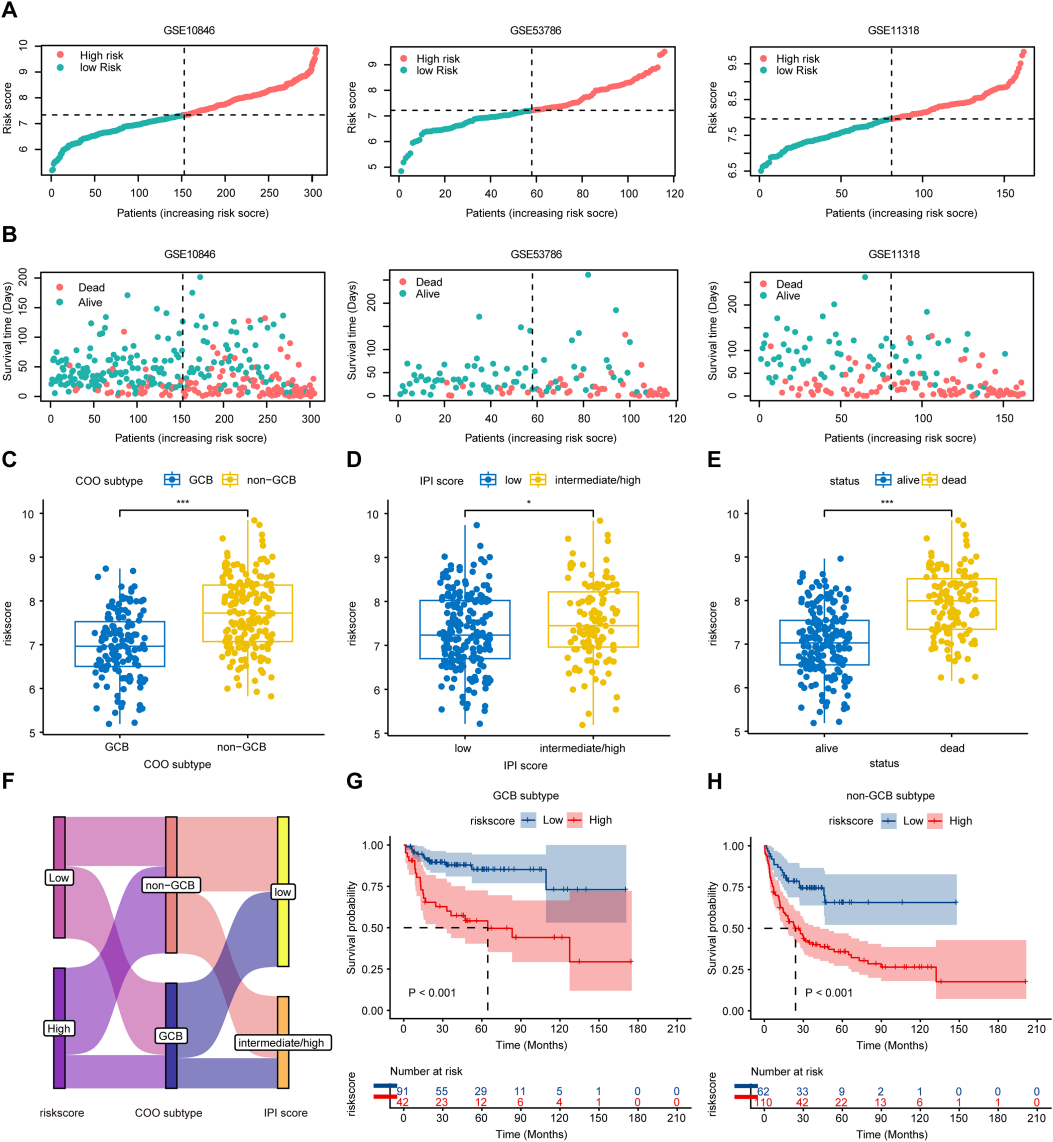
Clinical evaluation of the prognostic risk score. **(A)** Distribution of overall survival time in DLBCL patients. **(B)** Distribution of risk scores in DLBCL patients. **(C)** FAMscore in patients with GCB and non-GCB subtypes. **(D)** FAMscore in patients with low IPI scores versus intermediate/high IPI scores. **(E)** FAMscore in patients with alive versus dead OS status. **(F)** Sankey diagram illustrating relationships among risk score, IPI score, and cell-of-origin (COO) subtype. Bandwidth is proportional to the number of samples. **(G, H)** Kaplan-Meier survival analysis for patients with the GCB **(G)** and non-GCB **(H)** subtypes. *P< 0.05, ***P< 0.001.

The distribution of survival time and risk score showed that this signature effectively distinguished between high- and low-risk patients (**Figures 4A, B**). To further evaluate the clinical utility of the FAMscore, we investigated its relevance to clinicopathological features. Based on gene expression profiling, DLBCL can be classified into COO subtypes, namely the germinal center B-cell-like (GCB) subtype and the non-GCB subtype, which includes activated B-cell-like (ABC) and unclassified subtypes (32, 33). We observed that the GCB subtype, which is associated with a more favorable prognosis, exhibited markedly lower FAMscores (**Figure 4C**). IPI, a widely used prognostic tool for lymphoma that incorporates age, Ann Arbor stage, performance status, number of extranodal sites, and LDH level, was also examined. Patients with intermediate/high IPI scores had substantially higher FAMscores compared to those with low IPI scores (**Figure 4D**). Similarly, deceased patients also showed elevated FAMscores (**Figure 4E**). These findings further support the association between high FAMscore and adverse clinical outcomes.

To assess the prognostic value of the FAMscore within DLBCL subtypes, we performed survival analysis in GCB and non-GCB subgroups separately. The results indicated that in both subtypes, patients in the high-FAMscore group had significantly lower survival rates than those in the low-FAMscore group (**Figures 4F–H**). Furthermore, the FAMscore effectively stratified patients into distinct risk categories not only among those with low IPI scores but also in the intermediate/high IPI group (**Supplementary Figures 2A, B**), suggesting its broad applicability. To account for potential confounding by age, we conducted survival analyses in patients aged ≤60 years and those >60 years. The results confirmed that the FAMscore retained strong predictive performance across both age groups (**Supplementary Figures 2C–E**).

### 3.4 Establishment of a prognostic nomogram

**Figure 5:**
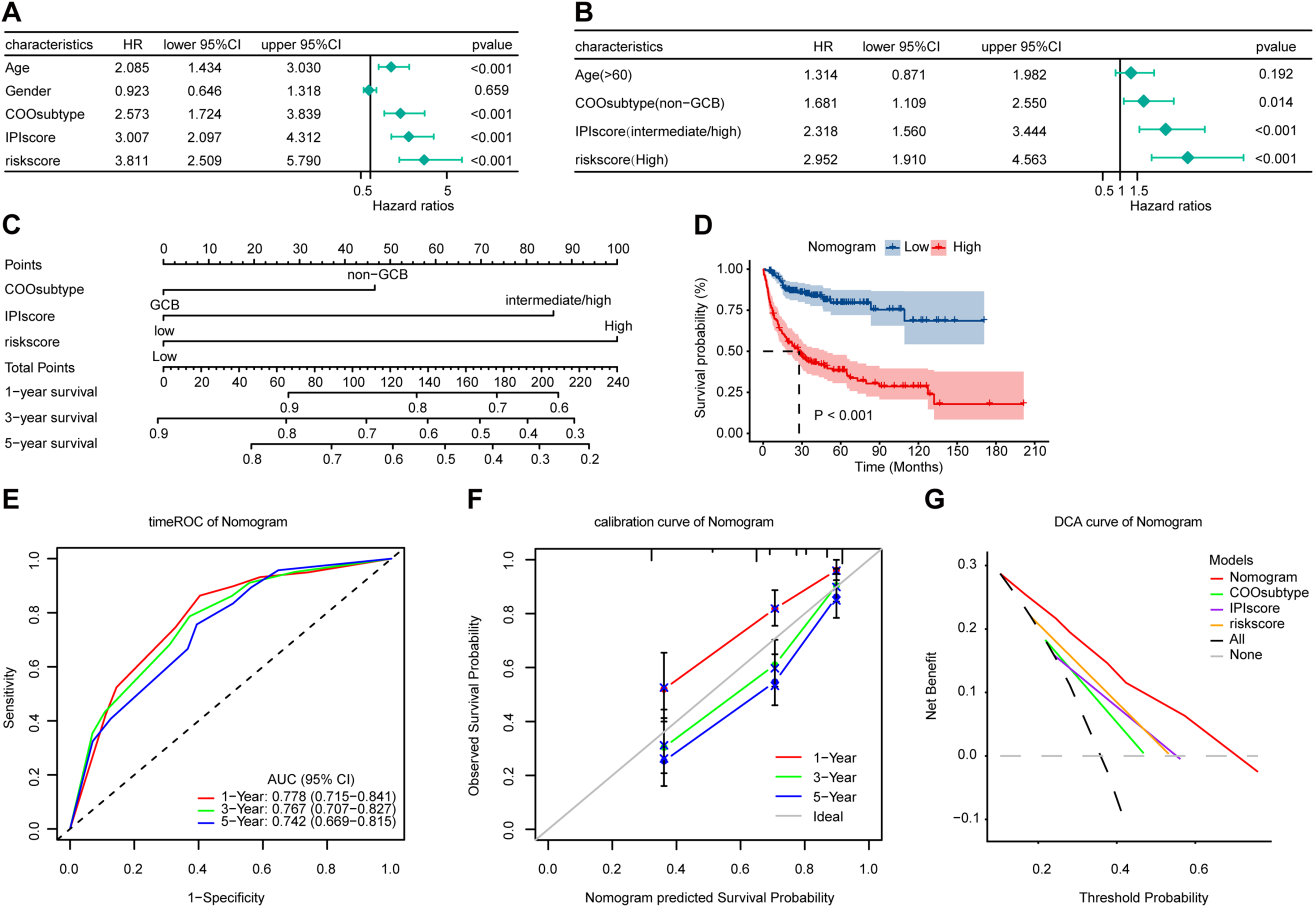
Establishment of a nomogram. **(A)** Univariate Cox regression analysis of the risk score and other clinical factors in the GSE10846 cohort. **(B)** Multivariate Cox regression analysis of the risk score and other clinical factors in the GSE10846 cohort. **(C)** Scoring System of the nomogram. **(D)** Kaplan-Meier survival analysis stratified by the nomogram score in the GSE10846 cohort. **(E)** Time-dependent ROC analysis of the nomogram in the GSE10846 cohort. **(F)** Calibration curve for assessing the nomogram’s predictive accuracy in the GSE10846 cohort. **(G)** Decision curve analysis (DCA) of the nomogram in the GSE10846 cohort.

To investigate the significance of the risk score and common clinicopathological features, univariate and multivariate Cox regression analyses were performed on the training cohort. The results showed that COO subtype, IPI score, and the FAMscore were independent prognostic factors for DLBCL patients (**Figures 5A, B**). This finding was further validated in external cohorts, where the FAMscore remained a significant predictor for overall survival (**Supplementary Figure 3**). These factors were subsequently integrated to construct a nomogram for quantitatively predicting survival probability in individual patients (**Figure 5C**). Survival analysis demonstrated that patients with higher nomogram scores had significantly poorer overall survival outcomes (**Figure 5D**). Time-dependent ROC curves and calibration curves indicated that the nomogram exhibited favorable sensitivity, specificity, and good agreement between predicted and observed outcomes (**Figures 5E, F**). Decision curve analysis further revealed that the nomogram provided a superior net benefit compared to any single indicator, while the FAMscore alone offered a net benefit comparable to that of the IPI score (**Figure 5G**). Importantly, when the same nomogram scoring system was applied to the GSE53786 and GSE11318 cohorts, the scores consistently correlated with overall survival, confirming the robust predictive capacity of the nomogram across independent datasets (**Supplementary Figure 4**).

### 3.5 The FAMscore is associated with the tumor immune microenvironment

**Figure 6:**
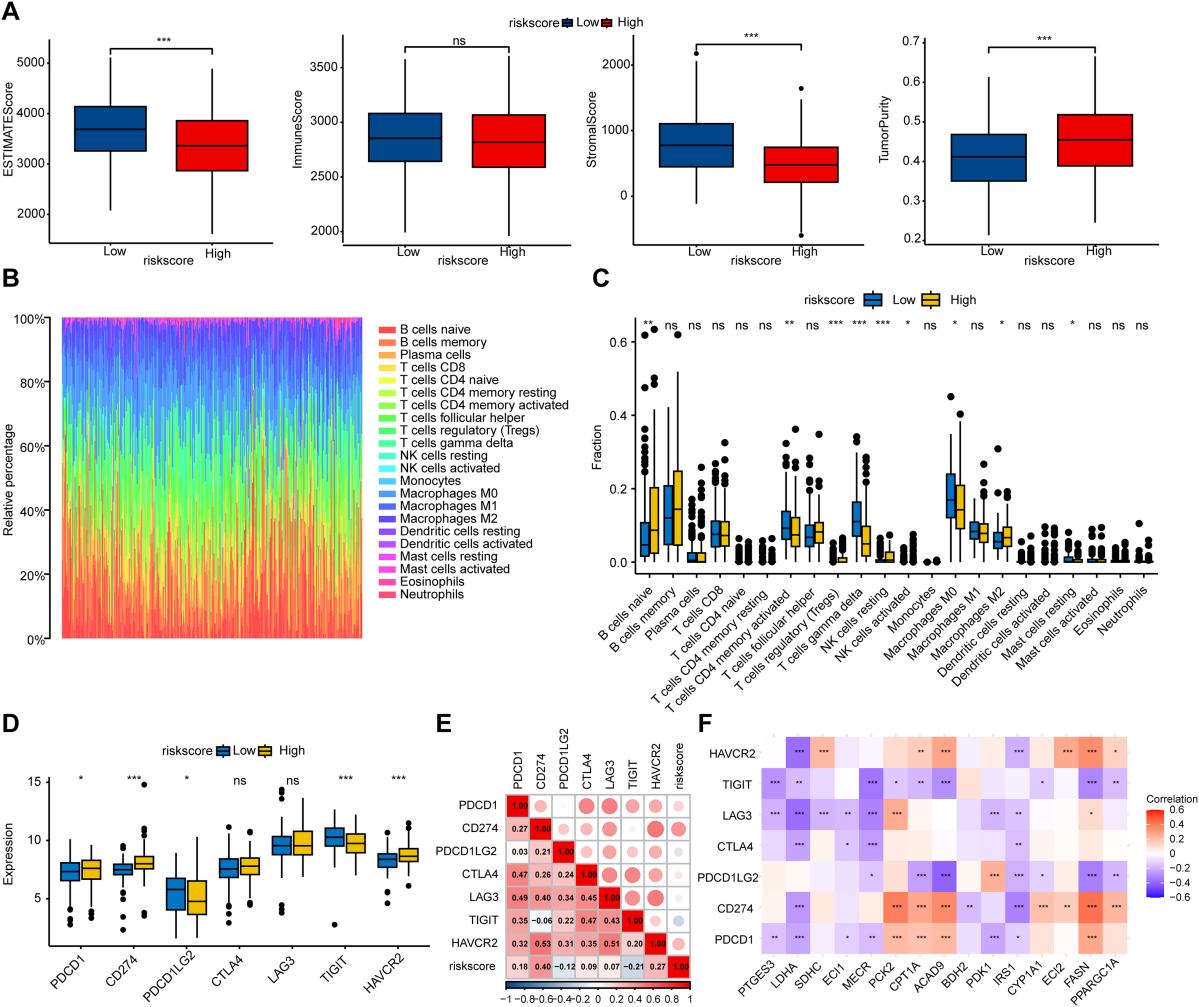
Characterization of the immune landscape in the low- and high-FAMscore groups. **(A)** Evaluation of the immune score, stromal score, and tumor purity using the ESTIMATE algorithm. **(B)** The composition of 22 immune cell subpopulations within the tumor microenvironment. **(C)** Abundance of infiltrating immune cells in the low- and high-FAMscore groups. **(D)** Expression of immune checkpoint genes in the low- and high-FAMscore groups. **(E)** Correlation analysis between the FAMscore and immune checkpoint gene expression. **(F)** Correlation analysis between the FMGs included in the FAMscore and immune checkpoint gene expression. *P< 0.05, **P< 0.01, ***P< 0.001.

We further evaluated the impact of the FAMscore on the tumor immune microenvironment. We first employed the estimate R package to calculate the immune score, stromal score, and tumor purity of DLBCL samples (**Figure 6A**). The results indicated that the high-FAMscore group exhibited higher tumor purity but lower stromal content. Next, the CIBERSORT algorithm was used to assess the proportions of 22 types of immune cells within the tumor microenvironment (**Figures 6B, C**). Marked differences in immune infiltration were observed between the two groups. The high-FAMscore group showed reduced abundances of activated memory CD4 T cells and gamma delta T cells, which is unfavorable for the formation and maintenance of immunological memory, weakens helper functions, and diminishes the capacity to kill tumor cells. Conversely, increases in regulatory T cells (Tregs) and M2 macrophages were noted, suggesting that a high FAMscore is associated with an immunosuppressive microenvironment. Furthermore, we examined the correlations between the FAMscore or the FMGs included in the FAMscore and the expression levels of seven immune checkpoints (**Figures 6D–F**). The FAMscore was positively correlated with PD-1, PD-L1 and TIM-3, but negatively correlated with PD-L2 and TIGIT. This suggests that patients in the high-FAMscore group may exhibit better responsiveness to immune checkpoint blockade therapy targeting PD-1, PD-L1, and TIM-3.

### 3.6 Immune pathway suppression in tumors with high FAMscore

To explore the mechanistic link between fatty acid metabolism and tumor immunity, we performed differential expression analysis between FAMscore-stratified risk groups, followed by gene set enrichment analysis (GSEA). The results revealed significant downregulation of several immune-related pathways in the high-risk group, including the interferon-γ (IFNγ) response, inflammatory response, and complement pathways (**Supplementary Figure 5**). IFNγ, a core effector molecule in antitumor immunity, is primarily secreted by activated T cells and natural killer (NK) cells. The downregulation of this pathway in the high-risk group suggests impaired effector T cell function within the tumor, leading to reduced IFNγ production and disrupted downstream signaling. This consequently diminishes MHC class I molecule expression on tumor cells, thereby impairing antigen presentation. The loss of IFNγ signaling may also hinder effector cell recruitment while promoting the accumulation of immunosuppressive cells, such as Tregs and myeloid-derived suppressor cells (MDSCs) (34, 35). Meanwhile, downregulation of the inflammatory response pathway primarily dampens the sensing and effector functions of innate immunity and may impact the initiation of adaptive immunity. Specifically, insufficient inflammatory signals could impede dendritic cell maturation and macrophage polarization toward the pro-inflammatory, antitumor M1 phenotype (36, 37). Additionally, suppression of the complement pathway may reduce complement-mediated tumor cell killing and opsonophagocytosis, ultimately compromising immune surveillance (38). Collectively, these findings indicate that concurrent suppression of multiple immune-activating pathways in the high-risk group contributes to an immunosuppressive tumor microenvironment, potentially linking fatty acid metabolism remodeling to immune evasion and poor patient prognosis.

### 3.7 Validation of the expression levels of FMGs

**Figure 7:**
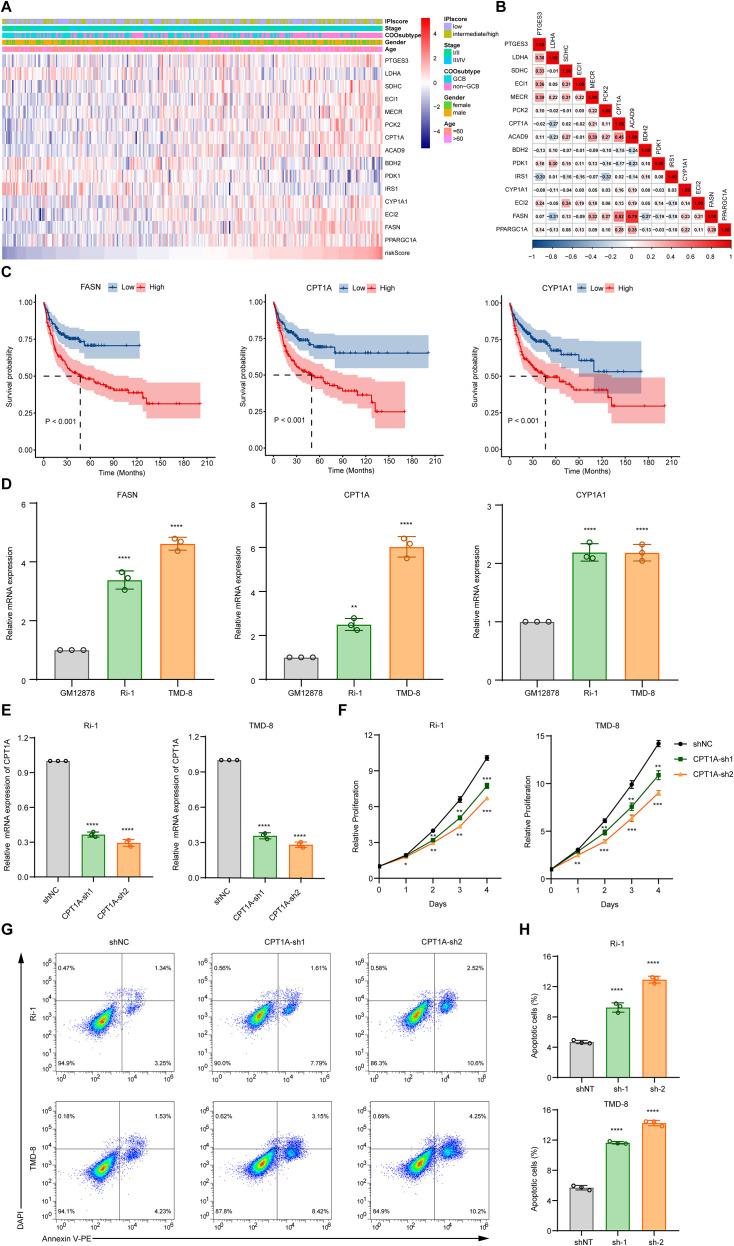
Study for FMGs included in the FAMscore. **(A)** Heatmap of FMG expression levels in the GSE10846 cohort. **(B)** Correlations among the FMGs in the GSE10846 cohort. **(C)** Kaplan-Meier survival analysis based on FASN, CPT1A, and CYP1A1 expression in the GSE10846 cohort. **(D)** Quantitative real-time PCR analysis of FASN, CPT1A, and CYP1A1 expression. Data are presented as mean ± s.d. and were analyzed by one-way ANOVA followed by Dunnett’s multiple comparison test. **(E)** Validation of CPT1A knockdown efficiency by qPCR. Data are presented as mean ± s.d. and were analyzed by one-way ANOVA followed by Dunnett’s multiple comparison test. **(F)** Assessment of cell proliferation using the CCK-8 assay. Knockdown of CPT1A significantly suppressed the proliferation of DLBCL cells. Data are presented as mean ± s.d. and were analyzed by two-way ANOVA followed by Dunnett’s multiple comparison test. **(G)** Representative flow cytometry images of apoptosis detected by Annexin V staining. CPT1A knockdown induced apoptosis in DLBCL cells. **(H)** Quantification of apoptotic cells. Data are presented as mean ± s.d. and were analyzed by one-way ANOVA followed by Dunnett’s multiple comparison test. *P< 0.05, **P< 0.01, ***P< 0.001, ****P< 0.0001.

We then validated the expression levels of the FMGs included in the FAMscore. The heatmap illustrates the expression patterns of the 15 FMGs relative to variations in the FAMscore (**Figure 7A**). Correlations among these genes are shown in **Figure 7B**. We performed survival analysis for the FMGs in the GSE10846 cohort, identifying FASN, CPT1A, and CYP1A1 as the three genes with the most significant prognostic value (**Figure 7C**, **Supplementary Figure 6**). Subsequently, we compared the expression levels of these three FMGs in the normal B-lymphoblastoid cell line GM12878 and the DLBCL cell lines Ri-1 and TMD-8 by qPCR (**Figure 7D**). Compared to normal B cells, the expression of FASN, CPT1A, and CYP1A1 was upregulated in DLBCL cells. Furthermore, analysis of single-cell RNA sequencing data also revealed increased FAMscores and elevated expression of these three FMGs in DLBCL samples compared to normal lymphoid tissues (**Supplementary Figure 7**).

### 3.8 Functional validation of CPT1A

Given that CPT1A is significantly upregulated in DLBCL cells compared to normal B cells, and its role in DLBCL progression remains poorly understood, we performed functional assays to assess its biological significance. CPT1A knockdown was achieved in two DLBCL cell lines, and the knockdown efficiency was confirmed by qPCR (**Figure 7E**). CCK8 assays showed that CPT1A downregulation significantly inhibited cell proliferation (**Figure 7F**). Furthermore, Annexin V staining revealed a marked increase in the proportion of apoptotic cells following CPT1A knockdown (**Figures 7G, H**). Collectively, these results demonstrate that CPT1A promotes the survival and proliferation of DLBCL cells.

## 4 Discussion

Under normal physiological conditions, fatty acid anabolism and catabolism maintain a dynamic equilibrium, collectively preserving fatty acid homeostasis. However, tumor cells exhibit a distinctive phenomenon characterized by enhanced fatty acid synthesis coupled with abnormally active catabolism to meet the demands of rapid proliferation, survival, and dissemination (39, 40). Numerous malignancies, such as lung, liver, breast, and prostate cancers, and precancerous lesions, undergo enhanced fatty acid biogenesis, providing abundant material and energy resources for malignant cells (41–44). Key enzymes involved in fatty acid synthesis are highly expressed in various tumors, with their activity closely correlated with tumor aggressiveness and poor prognosis (45, 46). Fatty acid oxidation (FAO) is also dysregulated in multiple human malignancies (47). Under conditions of hypoxia and glucose deprivation, tumor cells efficiently utilize FAO to generate ATP as an alternative energy source, facilitating survival in adverse microenvironments (48). Furthermore, the FAO process yields reduced coenzymes FADH_2_ and NADH, which help maintain redox homeostasis and enable tumor cells to counteract endoplasmic reticulum stress and mitochondrial dysfunction, thereby evading apoptosis (49, 50). The synergistic action of these pathways confers remarkable metabolic flexibility upon tumor cells, allowing them to adapt to the dynamically changing tumor microenvironment and acquire growth advantages. Lipid metabolic dysregulation has been demonstrated to participate in cancer cell proliferation, stemness maintenance, drug resistance, and metastasis (51, 52). Several studies have reported that targeting fatty acid metabolic pathways could induce cell death in DLBCL, suppress tumor progression, and ameliorate resistance to chemotherapy and immunotherapy (53–55). Nevertheless, prognostic models related to FAM in DLBCL remain to be thoroughly investigated.

In this study, we integrated multiple gene sets from the GO, KEGG, HALLMARK, and REACTOME collections within the MSigDB database to obtain a comprehensive list of FMGs. Classic DLBCL datasets were downloaded from the GEO database to serve as training and validation cohorts. These datasets comprise a substantial number of samples with relatively complete clinical information. Core FMGs with prognostic significance were screened using univariate Cox regression analysis, WGCNA, and PPI networks. To develop a predictive model, we applied 101 machine learning algorithms for model fitting. This integrative pipeline leverages the strengths of diverse algorithms to enhance the model’s predictive performance and generalizability. The model demonstrating consistent performance across multiple datasets was selected based on the mean C-index, and risk scores for patients were subsequently computed. Survival analysis and ROC curves indicated that the constructed FAMscore effectively stratified patients into distinct risk groups, exhibiting strong accuracy and stable predictive performance.

We further evaluated the association of FAMscore with COO subtypes, IPI score, and patient survival status. The results revealed that patients with the non-GCB subtype or intermediate/high IPI scores (associated with poor prognosis) tended to have higher FAMscores, suggesting that an elevated FAMscore is correlated with adverse clinical outcomes. Univariate and multivariate Cox regression analyses confirmed that FAMscore serves as an independent prognostic indicator in DLBCL. The established nomogram incorporating COO subtype, IPI score, and FAMscore also demonstrated reliable predictive capability. However, this study has several limitations. Our analysis primarily relied on retrospective datasets, and prospective clinical cohorts are required to validate the model’s predictive value and assess its applicability across diverse patient populations. Moreover, further *in vitro* and *in vivo* experimental studies are needed to elucidate the role and underlying mechanisms of FAM in DLBCL.

FAM functions as a critical factor in shaping the tumor immune microenvironment and profoundly influences the efficacy of anti-tumor immune responses (56, 57). Tumor cells often exhibit high expression of fatty acid transport proteins, enabling substantial uptake and storage of lipids to support their rapid proliferation. Altered fatty acid partitioning and local depletion of essential metabolites impair the function of effector T cells, leading to their exhaustion (58, 59). Lipid signaling molecules released by tumor cells can induce macrophage polarization toward an immunosuppressive M2 phenotype and recruit more Tregs into the tumor microenvironment (60, 61). These cell populations are well-adapted to survive in the nutrient-deprived, acidic tumor milieu, thereby establishing an immunosuppressive niche (62). Consistently, our study observed that patients in the high FAMscore group showed reduced abundance of activated memory CD4 T cells and gamma delta T cells, along with increased infiltration of Tregs and M2 macrophages, which may collectively promote tumor immune escape. Furthermore, FAMscore was positively correlated with the expression of immune checkpoint genes such as PD-1, PD-L1, and TIM-3, potentially offering valuable insights for the selection of immunotherapy strategies in DLBCL patients.

The functional importance of the individual FMGs included the FAMscore is supported by extensive evidence. For instance, a recent study focusing on mitochondrial-related genes in DLBCL identified PCK2 as a key prognostic factor, demonstrating that its downregulation under low-glucose conditions suppresses tumor cell proliferation and induces apoptosis (63). Similarly, LDHA is highly expressed in DLBCL, and its knockdown promotes apoptosis while inhibiting tumor cell growth and migration (64, 65). In addition, polymorphisms in the CYP1A1 gene have been reported to be significantly associated with an increased risk of DLBCL (66, 67). FASN promotes DLBCL progression through multiple mechanisms, including enhancing eIF4B-dependent translation of oncogenic proteins and activating the receptor tyrosine kinase c-Met signaling pathway (68, 69). In line with these findings, our qPCR results showed that CPT1A is significantly upregulated in DLBCL cells compared to normal B cells. Furthermore, functional experiments revealed that CPT1A knockdown promotes apoptosis and suppresses the proliferation of DLBCL cells.

In summary, this study employed bioinformatic screening and machine learning algorithms to develop an FMGs-based signature. This model demonstrates a significant association with OS in DLBCL patients and enables effective prognostic assessment. Furthermore, we elucidated potential links between FAM and the tumor immune microenvironment, as well as immunotherapy response. These findings enhance the understanding of fatty acid metabolic reprogramming in DLBCL, identify key prognostic FMGs, and provide a foundation for personalized treatment strategies and translational clinical research.

## Data Availability

The datasets presented in this study are available in the Gene Expression Omnibus repository, with accession numbers GSE10846, GSE53786, GSE11318, and GSE182434.
