# Supplementary material for: Identification of a fatty acid metabolism-related gene signature for prognostic prediction and immune microenvironment characterization in diffuse large B-cell lymphoma
Source: Front Oncol. 2026 Apr 10;16:1798939. doi: 10.3389/fonc.2026.1798939 (PMC13106545; doi:10.3389/fonc.2026.1798939)
Supplement: Supplementary file 1 [file DataSheet1.docx]

**Supplementary Information**

- **Supplementary Figures 1-7**
- **Supplementary Methods**

**
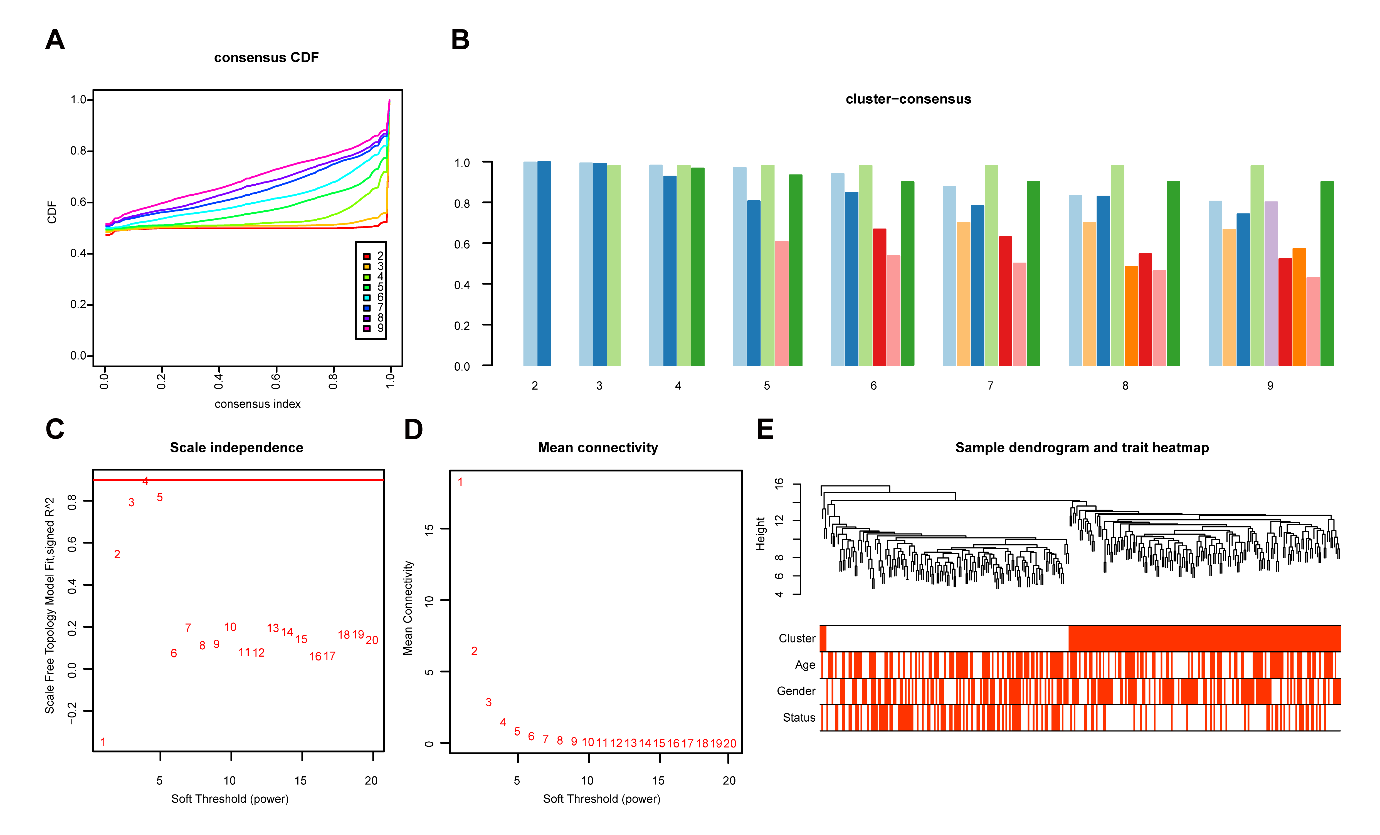
**

**Supplementary Figure 1.** Determination of the optimal k value for consensus clustering and the soft threshold β for WGCNA. (**A**) Cumulative distribution function (CDF) curves of the consensus matrix for k ranging from 2 to 9. (**B**) Cluster consensus across different k values. (**C**) Analysis of the scale-free topology fit index across various soft-thresholding powers. (**D**) Analysis of mean network connectivity across different soft-thresholding powers. (**E**) Sample dendrogram constructed using the optimal soft-thresholding power.


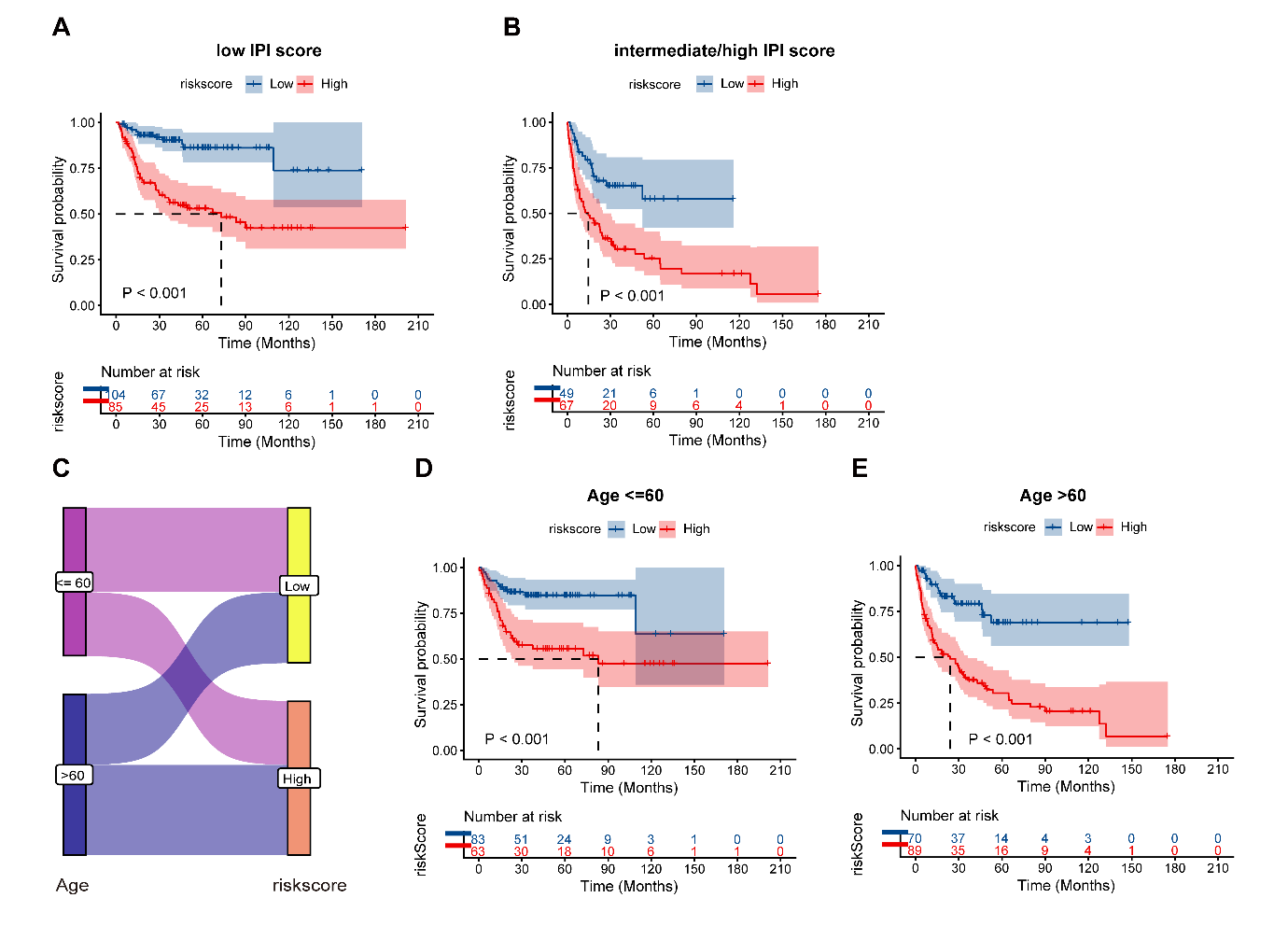


**Supplementary Figure 2.** Clinical implications of the FAMscore. (**A**) Kaplan–Meier survival analysis for patients with low IPI scores. (**B**) Kaplan–Meier survival analysis for patients with intermediate/high IPI scores. (**C**) Sankey diagram illustrating relationships between the risk score and age. **(D**) Kaplan–Meier survival analysis for patients aged ≤60 years. (**E**) Kaplan–Meier survival analysis for patients aged >60 years.


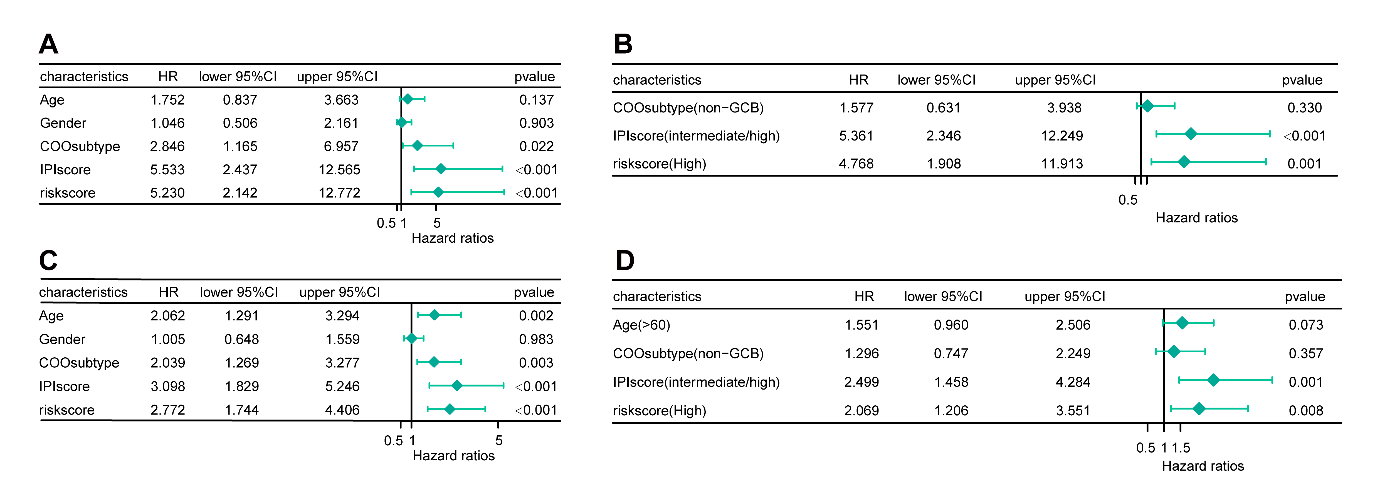


**Supplementary Figure 3.** Forestplots of the FAMscore and other clinical factors. (**A**) Univariate Cox regression analysis of the risk score and other clinical factors in the GSE53786 cohort. (**B**) Multivariate Cox regression analysis of the risk score and other clinical factors in the GSE53786 cohort. (**C**) Univariate Cox regression analysis of the risk score and other clinical factors in the GSE11318 cohort. (**D**) Multivariate Cox regression analysis of the risk score and other clinical factors in the GSE11318 cohort.


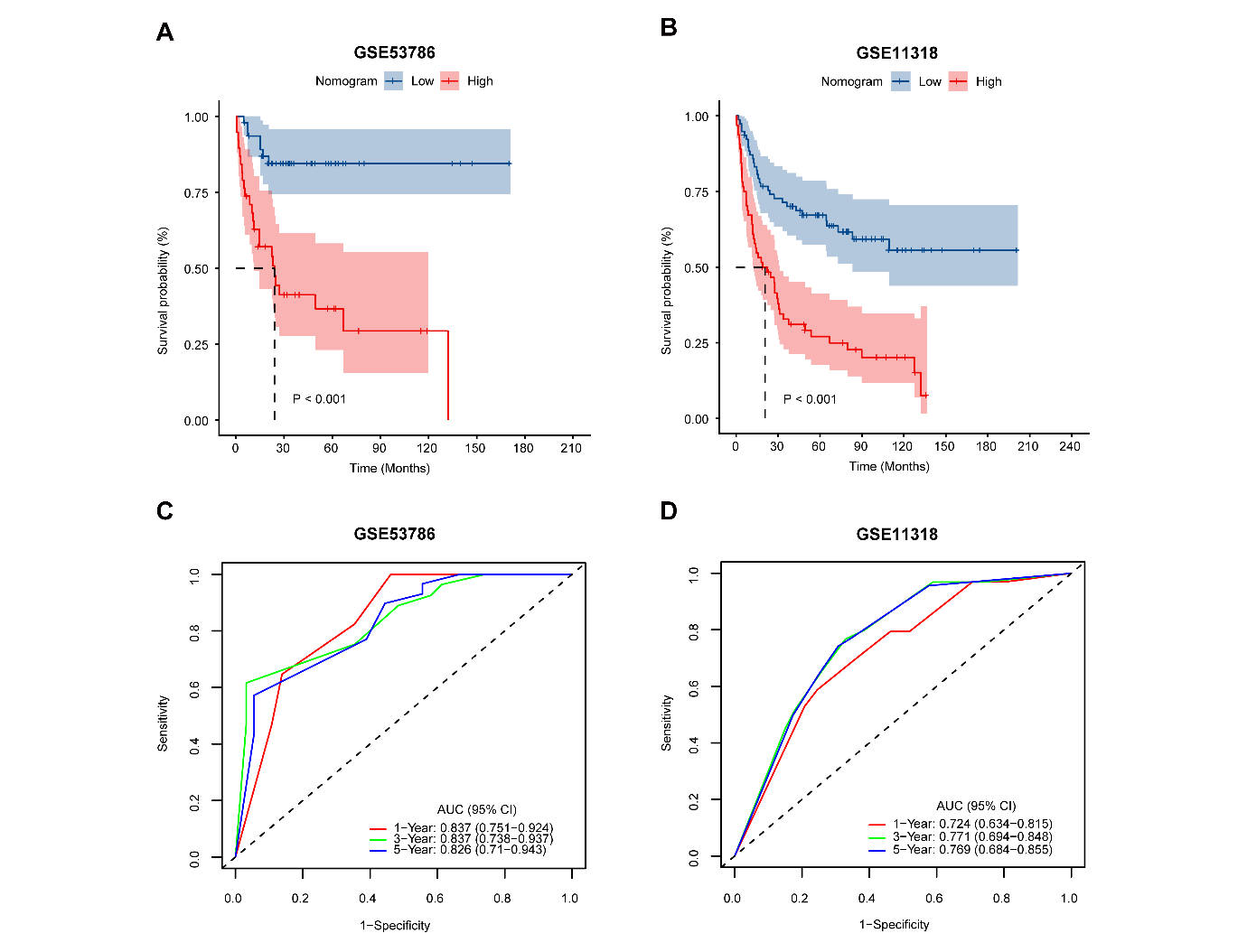


**Supplementary Figure 4.** Validation of the predictive capability of the nomogram. (**A**) Kaplan-Meier survival analysis based on the nomogram score in the GSE53786 cohort. (**B**) Kaplan-Meier survival analysis based on the nomogram score in the GSE11318 cohort. (**C**) Time-dependent ROC analysis of the nomogram in the GSE53786 cohort. (**D**) Time-dependent ROC analysis of the nomogram in the GSE11318 cohort.


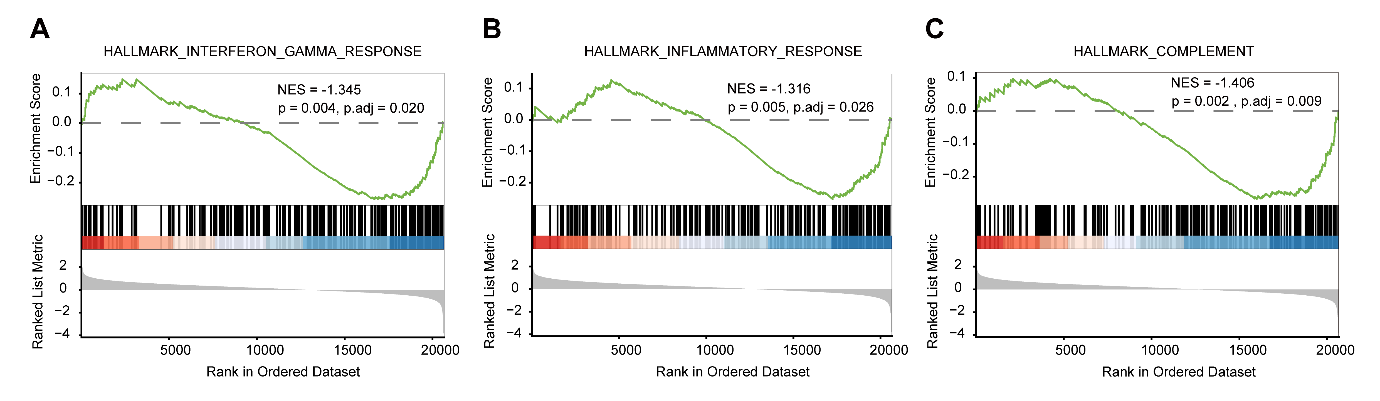


**Supplementary Figure 5.** Gene set enrichment analysis (GSEA) shows significant downregulation of several immune‑related pathways in tumors with high FAMscore. (**A**) Enrichment plot for the interferon-γ response gene set. (**B**) Enrichment plot for the inflammatory response gene set. (**C**) Enrichment plot for the complement gene set.


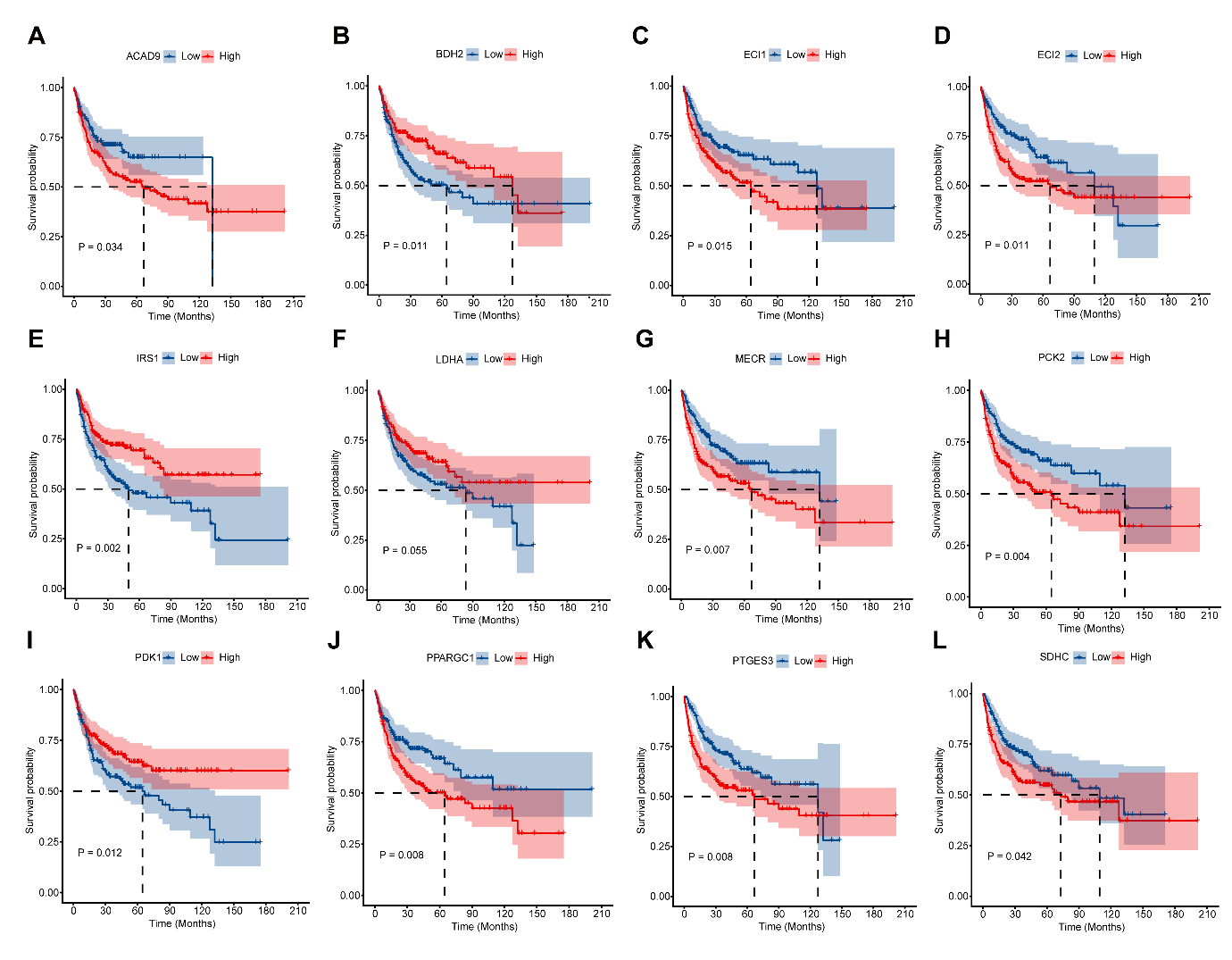


**Supplementary Figure 6.** Survival analysis for the FMGs.

Kaplan-Meier survival analysis based on ACAD9 (A), BDH2 (B), ECI1 (C), ECI2 (D), IRS1 (E), LDHA (F), MECR (G), PCK2 (H), PDK1 (I), PPARGC1 (J), PTGES3 (K) and SDHC (L) expression in the GSE10846 cohort.


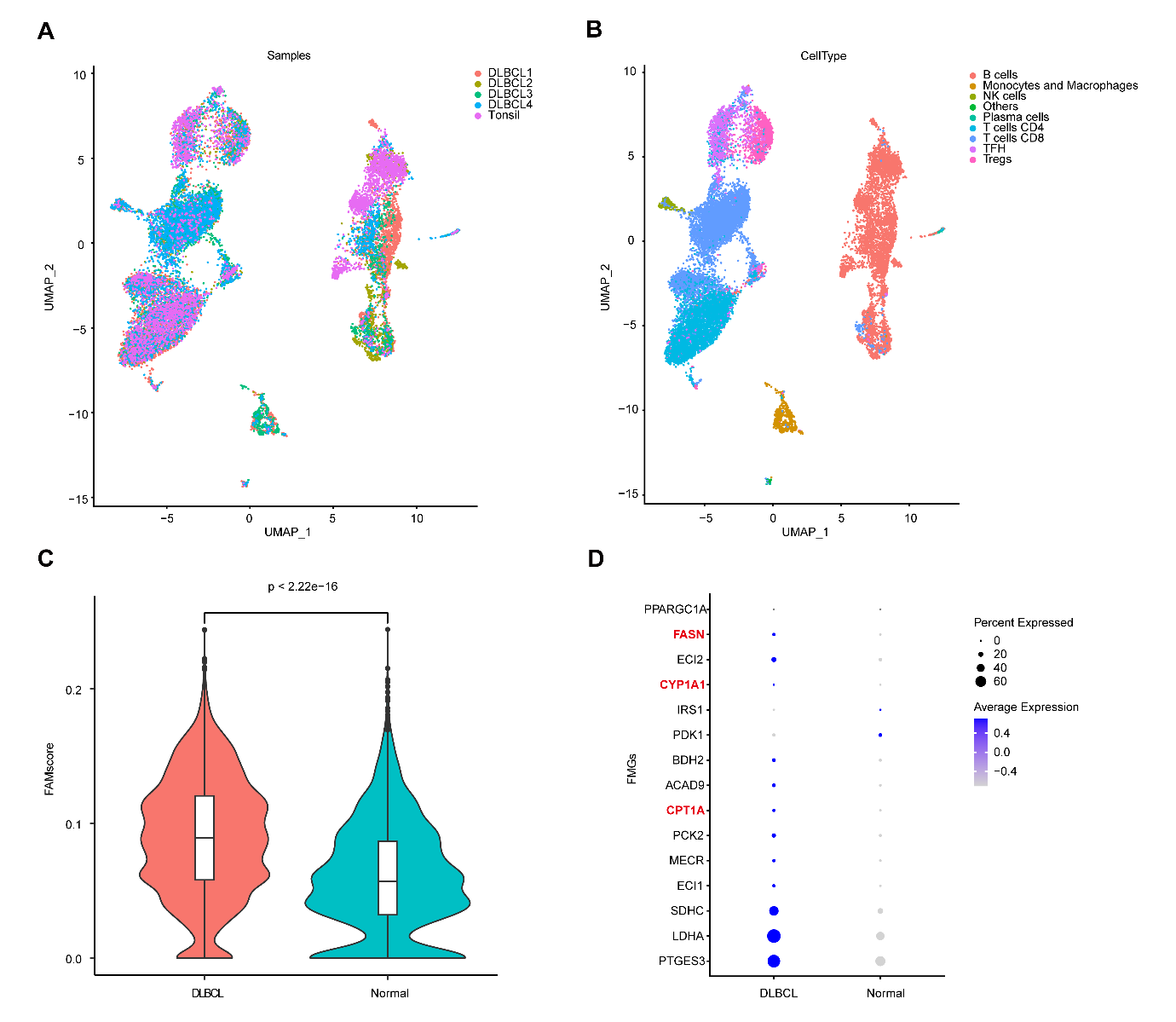


**Supplementary Figure 7.** Analysis of single-cell RNA sequencing data from the GSE182434 dataset. (**A**) UMAP plots of single-cell RNA sequencing data, colored by sample source. (**B**) UMAP plot of single-cell RNA-seq data, colored by cell type. (**C**) Comparison of FAMscores between DLBCL and normal tonsil samples. (**D**) Comparison of FMG expression levels between DLBCL and normal tissue samples.

**Supplementary Methods**

**Development of prognostic models using machine learning algorithms**

To develop a prognostic model with high accuracy and stability, we integrated 10 machine learning algorithms, including Elastic Net (Enet), Lasso, Ridge, Stepwise Cox, CoxBoost, Random Survival Forest (RSF), Partial Least Squares Regression for Cox Models (plsRcox), Supervised Principal Component Analysis (SuperPC), Generalized Boosted Regression Modeling (GBM), and Survival Support Vector Machine (survival-SVM). Among these algorithms, Lasso, Enet, stepwise Cox regression, and CoxBoost possess feature selection capabilities by shrinking the coefficients of irrelevant variables to zero or directly excluding them. RSF and GBM can output variable importance rankings, quantifying each feature's contribution to prediction. Ridge, plsRcox, SuperPC, and survival-SVM are primarily used for dimension reduction and prediction. In the GSE10846 cohort, we employed 101 algorithm combinations to fit predictive models under a 10-fold cross-validation framework.

The Enet, Lasso, and Ridge regression models were fitted using the glmnet R package. The regularization parameter λ was selected via 10‑fold cross‑validation using the cv.glmnet function, while the mixing parameter α (which controls the L1‑L2 trade‑off) was set to values ranging from 0 to 1 in increments of 0.1. The stepwise Cox model was built using the survival R package. Starting from a full Cox proportional hazards model, stepwise variable selection based on the Akaike information criterion (AIC) was performed using the step function. Three directional modes were considered: “both”, “backward”, and “forward”, each evaluated separately. The CoxBoost model was fitted via the CoxBoost R package, which applies component‑wise likelihood‑based boosting to a Cox model. The optimal penalty parameter (amount of shrinkage) was first determined using the optimCoxBoostPenalty function. Subsequently, the optimal number of boosting steps was selected by 10‑fold cross‑validation via the cv.CoxBoost function, and the final model was obtained using the CoxBoost function with the chosen penalty and step number. The RSF model was implemented via the randomForestSRC R package. The number of trees (ntree) was set to 1000, and the node size (nodesize) was set to 2. The splitting rule used was log‑rank, and variable importance and proximity were computed. The plsRcox model was implemented with the plsRcox R package. The cv.plsRcox function was used to determine the optimal number of components, and the plsRcox function was then applied to fit the model. The SuperPC model was built using the superpc R package. The supervised principal components were derived from the training data, and the optimal feature score threshold was selected by 10‑fold cross‑validation via the superpc.cv function. The GBM model was fitted with the gbm R package. An initial model with 10‑fold cross‑validation was run over 10,000 trees to determine the optimal number of boosting iterations (i.e., the number minimizing the cross‑validated error). A final model was then refitted using that optimal number of trees via the gbm function. The survival‑SVM model was implemented via the survivalsvm R package. The regression variant of survival support vector machines was employed, which adapts inequality constraints to handle censored data. The model was trained using the inverse probability of censoring weighting approach, with the parameter gamma.mu set to 1 and the optimizer method set to "ipop" (interior point method).
